# A qualitative study of the barriers to utilizing healthcare services among the tribal population in Assam

**DOI:** 10.1371/journal.pone.0240096

**Published:** 2020-10-08

**Authors:** Bandita Boro, Nandita Saikia

**Affiliations:** Centre for the Study of Regional Development, Jawaharlal Nehru University, Delhi, India; University of Western Australia, AUSTRALIA

## Abstract

**Objective:**

We aim to explore the barriers to accessing modern healthcare services in two tribal populations in Assam.

**Methods:**

In March 2018, we conducted qualitative research through 60 in-depth interviews with men and women aged 15 to 50 from Bodo and Rabha tribes in Udalguri and Baksa districts of Assam. We interviewed a group of health-service providers from public health facilities to understand the demand-supply balance in those facilities.

**Findings:**

On the demand side, direct and indirect financial obstacles, distance to health facilities, poor public transportation, perceived negative behavior of hospital staff, and lack of infrastructure were the main barriers to utilizing healthcare facilities. On the supply side, doctors and nurses in government health facilities were overburdened by demand due to a lack of human resources.

**Conclusions:**

Our study highlights the barriers to utilizing health facilities; these are not always driven by factors linked to the patient’s socio-economic status but also depend significantly on the quality of the health services and other contextual factors. Although the government has made efforts to improve the rural healthcare system through national-level programs, our qualitative study shows that these programs have not been successful in enhancing the rural healthcare system in the study area.

## Introduction

Tribal people are diverse populations and live in varied local environments and nations, with important implications for their health [1]. The tribal or Adivasi population in India constitutes a total population of 104 million, i.e., 8.6 percent of the Indian population, which makes the world’s largest population of indigenous people [2]. International studies provide evidence that tribal populations have poorer health and social outcomes than non-tribal populations [3]. India’s indigenous or tribal people too exhibit poorer health and lower social indicators than the general population [4, 5]. Further, these indicators vary widely within these populations across regions [6].

Previous quantitative studies suggest that even with the remarkable achievements of modern medicine, healthcare delivery services in tribal communities still lack [7–9]. The challenges in accessing modern healthcare services and the unique ways in which tribal people seek health care are the main issues in the area of tribal health [8]. Tribes are still dependent on traditional healthcare practices prescribed by “traditional healers,” even if modern healthcare facilities are available [10, 11]. Traditional healers are found mainly in rural India and frequently have no professional qualifications. They provide local remedies that they learn by being apprenticed to other traditional healers [12]. There is a debate on whether there is tribal resistance to modern medical practices or the tribal people are becoming more inclined toward modern medicine.

It is worth noting that Assam has the highest level of infant, maternal, and adult mortality in India [13–15]. It is also one of the most economically least developed Indian states [16] and a state with one of the lowest ranks in the Human Development Index [17]. The tribal population comprises nearly 12.4% of Assam’s total population, with 23 distinct tribal groups, of which nine are major tribal groups [2].

**Fig 1:**
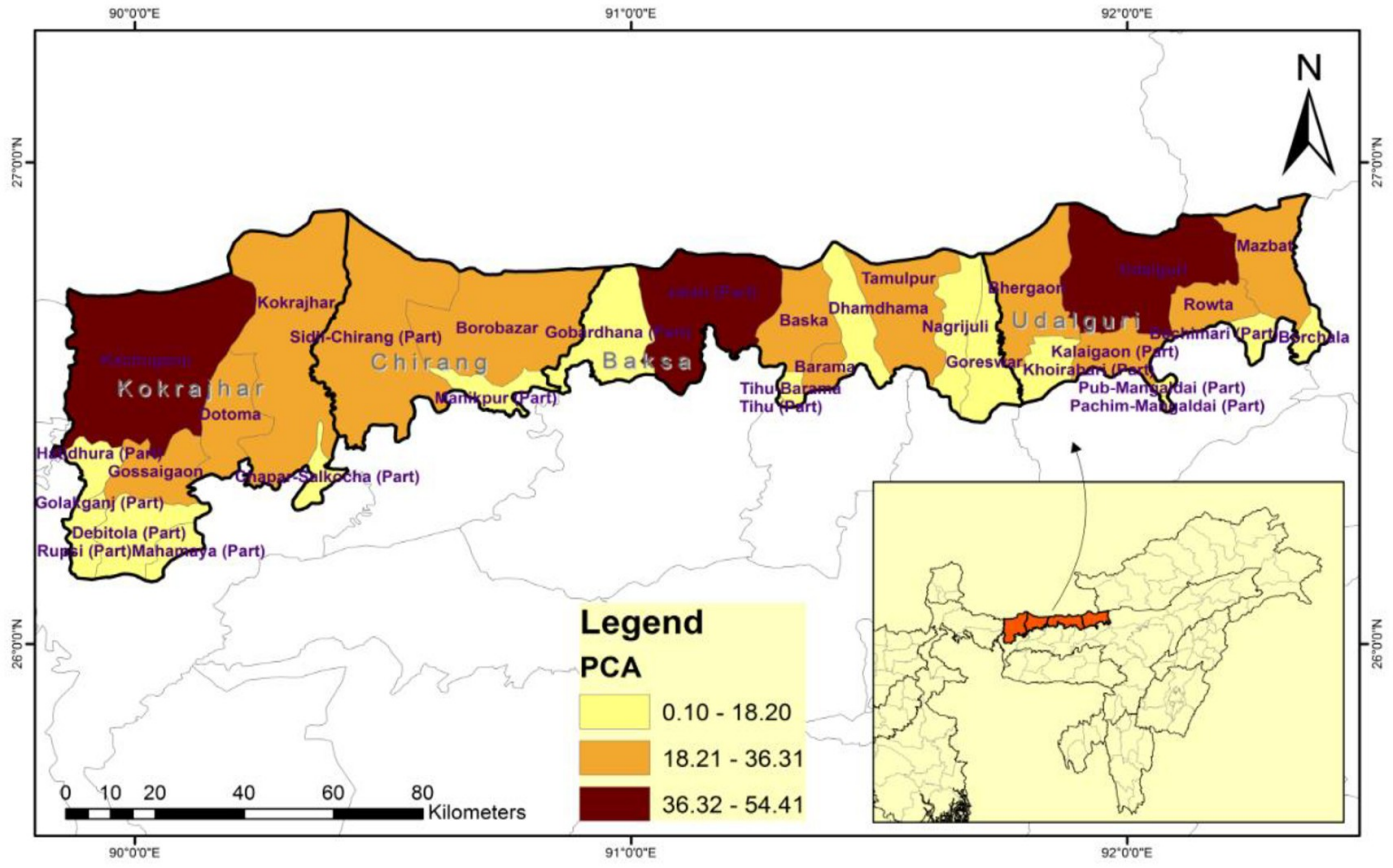
Health infrastructure index in BTAD of Assam, Census of India, 2011.

Our study area is the Bodoland Territorial Area Districts (BTAD), a least-developed area of India. This region is one of the most underdeveloped and isolated areas, not only in India but also in Assam. The leading tribal group of this area, known as “Bodo,” has a long history of armed revolt based on demands for their homeland, which ultimately led to the establishment of BTAD in 2003 within the political boundaries of Assam. (Fig 1). Despite the area having an autonomous tribal council, the tribal communities in the study area have poorer social and economic indicators.

Udalguri and Baksa district of BTAD is a remote area with poor transport and communication infrastructure. Public health facilities are predominant health service providers. The district’s hospitals and other subdivision hospitals are located at least 20–25 km from the villages. Primary health centres (PHC), state dispensaries, sub-centres, and community health centres (CHC) are available at a distance of 5–15 km, but the facilities provided by those are not sufficient. The services offer primary care, preventive services, and maternity care (including normal delivery). Services at the public health facilities are staffed by doctors, nurses, midwives (usually responsible for primary maternity care, including antenatal care (ANC) and normal delivery), medical assistants, and pharmacists. When we conducted the study, a full-time doctor or staff was not available in all public health facilities. There are also a small number of private clinics operating in the nearby towns located at a distance of 10-15km.

Previous quantitative studies fail to put the lower utilization of modern healthcare services among the tribal population in India into context [18]. In contrast, qualitative methods can investigate the context of underutilization of health services by incorporating open questions and opportunities to provide views on issues such as the quality of care within services and obstacles to access. Qualitative research uses a variety of techniques that can elicit information of relevance both to patients and service providers. This study aims to examine the barriers to healthcare service utilization among the tribal population in Assam.

## Data and methods

### Sample

A sample of 60 individuals (men and women) belonging to any tribal community in the study area participated in the survey. Out of the four districts of BTAD, we randomly chose two (Udalguri and Baksa), from which we selected six community developmental blocks (district subdivisions). To choose the blocks, we first calculated an index of health infrastructure using a combination of health infrastructure and health professional data (listed in S1 Appendix, source of data: Primary Census Abstract 2011, India) related to 37 blocks in all four districts of Kokrajhar, Baksa, Chirang and Udalguri of BTAD. We divided the health infrastructure index value into three categories: high-, medium-, and low-development zones. We then chose one block from each category in the two selected districts. Finally, we selected Udalguri, Rowta, and Khoirabari blocks from Udalguri district; and Jalah, Tamulpur, and Goreshwar blocks from Baksa district.

Two villages were selected randomly from each block, one having a public health facility and one without. We used snowball sampling to identify respondents who belonged to any tribal community. The final sample consisted of people mainly from two tribes, i.e., Rabha and Bodo.

### Study participants and methods

In total, 60 qualitative in-depth interviews were conducted, comprising 40 health service users (24 women and 16 men) and 20 health professionals. The inclusion criteria for participation included: any tribal men or women aged 25–50, residing in the study setting, and have consented to participate.

We interviewed a group of health-service providers from rural public health facilities in the study area to gain insights into the demand-supply balance in healthcare services in the study area. These health professionals were doctors, nurses (Auxiliary Nurse Midwife [ANM] and General Nursing and Midwife [GNM]), Accredited Social Health Activists [ASHA]), medical officers, and medical technicians at the healthcare facilities.

A semi-structured open-ended questionnaire was prepared after reviewing previous studies [19–21]. The service users were asked about their socio-economic background, any health problems, choice of health provider, any obstacle in accessing healthcare services, belief in traditional healers, doctors’ and nurses’ behavior at the health facilities, counseling by medical staff at antenatal visits, and the environment of the health facilities. The service providers were asked about the various issues and challenges involved in delivering healthcare services. Questions were asked in the Assamese language. The interviews were audio-recorded with a duration of twenty to forty-five minutes. The interviews were conducted in March 2018. Pseudonyms have been used for all participants to protect their privacy.

### Data collection

BB conducted interviews with the assistance of a local person who helped in accessing the study area. Informal verbal consent was obtained from the participants before conducting the interviews. BB went to any PHC, CHC, state dispensary, or a sub-center that was able to be accessed in the villages of the selected blocks. Any doctor or other medical staff who were available at the time of visits were invited to participate. Interviews were also conducted with the respondents who were also present in the health facility at that time after gaining permission from the doctor. In the villages where there was no health facility available, the researcher BB visited households with the village headman’s assistance and interviewed household members who consented to participate. Few participants refused to participate, citing their personal reasons and were hence excluded from the study. Notes were taken during the interviews to ensure that no essential points would be missed out in reporting.

#### Reflexivity

At the time of conducting the research, the interviewer, BB was an MPhil student. The interviewees belonged to the same tribe as that of BB. To build familiarity and rapport with the interviewees, BB first stressed her identity as belonging to the tribe. However, she could not speak the language of the Bodo tribe. During instances when the interviewee could not understand the researcher, the village headman or the local person helped to maintain seamless communication. The goals and objectives of the study were explained to them, and an assurance was given that the researcher has no personal ulterior motives for conducting the study. The researcher pre-assumed the health facilities of the area to be insufficient but was ultimately dependent on the responses and the narratives of the respondents.

### Methodology

This study is based on the narrative research approach, which relies on the spoken words of the interviewees. Analysis of the reports of this qualitative study is conducted using the guideline of consolidated criteria for reporting qualitative studies (COREQ) [22].

### Data analysis

Thematic analysis was used for this study. The typed transcripts were reviewed for accuracy, completion, and familiarization and were read by both authors. BB assigned preliminary codes, and then further categorization and sub-grouping was undertaken. The themes emerging from the codes were collated and discussed and agreed upon by both authors. NVivo 11 was used to assist in handling the data for analysis.

### Ethical considerations

Ethical approval was granted by the M.Phil./PhD committee review board of the Centre for the Study of Regional Development, School of Social Sciences, Jawaharlal Nehru University, New Delhi, India, with the office order no- 120/18 dated on 01-03-1018. Respondents’ verbal consent was gained before the interviews were conducted. The participants were mostly illiterate; hence it was decided that verbal consent would be obtained from all the participants. Prior permission for the audio recording of interviews was obtained from participants.

## Results

### Participant demographic characteristics

Table 1 shows the number of respondents interviewed by their different socio-economic characteristics. Most of the respondents were cultivators or agricultural laborers. The majority were Bodo tribes, and the rest were Rabha tribes in the region. The women and men interviewed were between the ages of 30 and 45 and currently married. More than half were Hindus, and the rest were Christians.

**Table 1:** Background characteristics of the respondents in the BTAD area of Assam, March 2018.

Background characteristics	%	N = 40	Background characteristics	%	N = 40
**Gender**			**Age**		
Male	40.00	16	26–30	15.00	6
Female	60.00	24	31–35	30.00	12
**Religion**			36–40	27.50	11
Hindu	77.5	31	41–45	20.00	8
Christian	22.5	9	46–50	7.50	3
**Type of tribe**			**Education**		
Bodo	75.00	30	No education	20.00	8
Rabha	25.00	10	Less than 8th standard	5.00	2
**Occupation**			8th to 11^th^	57.50	23
Cultivator	27.50	11	12^th^ or more years of education	17.50	7
Housewife	25.00	10	**Marital status**		
Small business	15.00	6	Unmarried	7.50	3
Teacher	5.00	2	Married	90.00	36
Anganwadi worker	7.50	3	Widowed	2.50	1
Informal Labourer	20.00	8	**Number of family members**		
			2–4	42.50	17
			5–8	52.50	21
			9 and above	5.00	2

In the sections that follow, we use illustrative quotations from the service users and service provider participants to describe the major themes identified at each level.

### Themes from qualitative analysis: Service users’ perspectives

#### Affordability

The majority of the participants stated that they could not afford the modern healthcare services available, either because of their high prices or because they had a low income or a combination of the two.

Yes, money is, of course, a problem for us to use healthcare services. Borrowing and sometimes selling our pigs or hens are the only ways to arrange money for treatment.

(Bijaya, 44-year-old woman)

Direct costs related to ensuring "good treatment" in private hospitals were also described as barriers to accessing healthcare services.

We are cultivators, and money is not always available. There is a private clinic which is 10kms from here, and the user fees are 1500–2000 rupees. So we cannot afford to go there even though we want to.

(Ajit, 38-year-old man)

Most participants acknowledged that living with non-communicable diseases (NCDs) or any other long-term disease places an extra financial burden on their household. Thus, people with a severe illness sometimes stop their treatment midway due to money concerns, irrespective of whether they were cured.

It has been ten years since I have been sick. I have already sold half of my land to cover my health expenses. That is why I have stopped seeking treatment.

(Jamuna, 49-year-old woman)

I have been suffering from joint pain for the past two years. I went to the Guwahati Medical College in Guwahati twice before. However, after that, I am no longer having treatment because of unaffordability, as my husband is a small farmer.

(Anima, 44-year-old woman)

In addition to the direct cost, many respondents reported that the indirect costs of a healthcare facility were also prohibitive. The primary concern was the expense of arranging transportation to the facilities.

We are informal laborers and earn only a meager income in a month. The hospitals are located at a distance of at least 10–20 km from our village. Since there is no public transport, we need to arrange a rented vehicle which costs around Rs. 500–600. So at times when we cannot arrange the money for the transport cost, we cannot visit the doctor.”

(Jiten, 35-year-old man)

#### Quality of care in the government health facilities

Good quality health care emerged as a second barrier to accessing health facilities. The issues included unavailability of doctors, difficulty in scheduling an appointment, and long waiting times at appointments.

Limited and irregular doctor’s availability in the government healthcare facilities was reported by most of the participants, which deterred them from visiting a healthcare facility.

Most of the time, the doctors are not available, and even at times of dire need, we do not get a doctor.

(Aruna, 35-year-old woman)

A husband (32 years old) took his pregnant wife to a nearby Primary Health Centre for a check-up, but the queue was too long for them to see the doctor. Ultimately, they had to visit a private clinic in a nearby town, which was almost 28 km from their home in an area with no proper roads and communication.

I took my wife for a check-up but could not even meet the doctor despite visiting continuously for three days. I requested a person I knew at the counter to arrange something, but that even didn’t help.

(Pranjit, 28-year-old man)

#### Poor quality of interpersonal care

Perceived disrespectful behavior or lack of interest in patients by the health care providers and nurses in private and public health care facilities was expressed by most of the respondents. The respondents spoke about rudeness on the part of doctors and nurses, mainly if the patient came from a poor economic background.

Seema (37-year-old woman) was suffering from weakness. Her husband was landless agricultural labour. She did not expect to be treated disrespectfully.

The doctor, after checking me, said that you do not have money on how you will pay for treatment. I felt so hurt and discouraged that I never dared to revisit a doctor.

#### Lack of medicines and other medical equipment

The majority of the participants expressed dissatisfaction with the availability of medication, which forced them to buy medicines at private facilities and pharmacies.

Only medicines for fever, cough, and cold are available in the nearby dispensary. Most of the time, the stock of medicines runs out, and it takes 3–4 days for new medications to arrive.

(Suren, 44-year-old man)

The participants reported a lack of equipment and resources at the government health facilities, which led to poor-quality care. People described the lack of equipment in services, especially in diagnostic services.

There is no facility for the treatment of major severe diseases in this hospital. Even for a CT scan or ultrasound, we need to go to a neighboring town that is quite far away. It would have been beneficial for poor people like us if there were treatments for all kinds of diseases locally.

(Niru, 34-year-old woman)

#### More dependence on traditional medicines

The study found that the tribal people are highly dependent on traditional medicines. Despite their socio-economic background, people use various traditional medicines prescribed by the traditional healers locally known as "*Ojha"* or "*Kabiraj*.”

Most of the study area participants reported an innate trust of traditional medicines while holding the belief that traditional medicines suit their bodies more than modern ones.

I am relieved when I use traditional medicine since I am accustomed to it. I have more faith in traditional medicines.

(Rabiram, 36-year-old man)

We have been using traditional medicines since birth, and we are used to it. Moreover, sometimes they are more helpful than modern ones.

(Niru, 34-year-old woman)

The *Ojha* and *Kabiraj* prescribe medicine at a much lower cost, making them easily affordable for those who were mainly agricultural laborers on a low income.

Traditional medicines for most illnesses like jaundice, fever, cough, cold, chickenpox, pneumonia can be purchased for a mere Rs 10–20, so we more often go for traditional medicines. We can even bargain about medicine prices and purchase them in any quantity we want.

(Jiten, 42-year-old man)

Due to the unavailability of doctors, local people prefer to go to traditional healers.

Due to the doctor’s unavailability at the required time, people in our villages go to the Ojha or Kabiraj, who are always available.

(Anjali, 31-year-old woman)

#### Low level of education and ignorance

Respondents’ narratives indicated that people with a low level of education (ten years or less of schooling) were unaware of the benefits of modern healthcare facilities.

A respondent named Meera was the 33-year-old mother of a four-year-old child. Her son had a bone fracture in his left hand while playing. Listening to other people’s advice, she took her son to an *Ojha* who put medicinal herbs in the boy’s hand and strapped it up; he assured the mother that there was no need to take her son to a doctor, as he would soon be cured. After 15 days, she took him to a private doctor who said that they had brought him too late, as the hand has been wrongly strapped up. She was advised that the boy would need surgery to repair the deformity.

I regret now for going to that Ojha and trusting him, and for that, I am paying the price now. It is because of my ignorance and lack of knowledge that I couldn’t make the decision myself and listen to other people. I will never go to Ojha or Kabiraj and even persuade other people not to do so.

#### Distance to the health facility

The distance to the health facility was cited as another barrier to access health care facilities.

Visiting the healthcare facilities is really a problem as they are located at least 15–25km from our village. Plus, there is no public transport in our area. Arranging a vehicle on our own is the only option for us as it is not possible to go on walking.

(Hima, 28-year-old woman)

### Barriers to accessing health facilities: Providers’ perspective

The poor quality of the government health facilities was the most critical barrier highlighted by the interviewees. To understand the reasons for such low-quality facilities, we undertook interviews with health personnel from different government health facilities in the area. The various themes that emerged from the interviews with health personnel are discussed below.

#### Overburdening of health facilities

The healthcare providers reported that a lack of human resources meant they were overburdened with patient numbers.

The number of staff in this model hospital is not enough. There are only three doctors in this hospital and two GNM nurses. More than 100 patients come every day, and thus we cannot give enough time to each patient.

(Doctor at a state dispensary, 38-year-old man)

#### Lack of education and patient non-compliance

The majority of medical personnel reported that most patients with low levels of education failed to cooperate with medical advice.

People believed more in traditional medicines than modern ones. They came to the doctor only when they failed to recover using the medicines from *Ojha* and *Kabiraj*.

I have seen and experienced in the last ten months that most people go for traditional medicines first and then come to us only if they do not get cured.

(Doctor at a CHC, 30- year-old man)

It was perceived that people with low levels of education had misconceptions about certain medicines and tests.

When iron tablets are prescribed to pregnant women, they do not take them. They think it will increase the weight of the baby. Some say they get a particular smell, so they cannot take those tablets.

(ANM nurse at a sub-center, 35-year-old woman)

The patients are not educated, and so they cannot easily grasp the things we say. People hesitate to visit us. Even when people are educated, they are not wise. There is a significant communication gap between us.

(Doctor at a state dispensary, 37- year-old man)

However, most health personnel reported increasing use of health facilities by tribal people in recent times.

In the past few years, the utilization of modern health facilities has increased among tribal people due to awareness programs and counseling though it is still unsatisfactory.

(a doctor at a PHC, 50-year-old man)

#### Poor transport and communication

The medical respondents reported transport and communication as being the primary barrier to fulfilling their duties. The villages in the area are so isolated that no proper roads are connecting them.

We need to visit door-to-door houses for treating pregnant women and immunizations. It becomes challenging to visit the houses and give treatment during the monsoon season because of floods. The poor road network is a significant challenge to performing our duties.

(ANM nurse of sub-center, 40-year-old woman)

Distances were also a problem in the area. The public health facilities were located very far from the villages, at least 10–15 km. Lack of public transport further added to the problem.

If a patient has an emergency at night, he/she will not be coming to the hospital because of the distance. He or she will wait until the next morning. Moreover, they find difficultly in arranging a vehicle at night.

(Doctor at a CHC, 37-year-old man)

#### Lack of government intervention

Lack of government support is one of the main problems highlighted by public health facilities’ health personnel.

The CHC in our area is not well maintained, and there is much scope for improvement. There are no proper diagnostic facilities here. Even there is a shortage of doctors here. If the government checks these loopholes correctly, then the problem of a proper health facility will be solved.

(Doctor at PHC, 50-year-old man)

## Discussion

The present qualitative study among the tribal population of the Baksa and Udalguri districts of Assam explored different barriers to accessing healthcare facilities. In general, the barriers are due to socio-economic and infrastructural factors.

In a tribal area, for every 3000 populations, there is one sub-center, a primary health center for every 20,000 population, and a community health center for 80,000 populations. Health workers like the doctors and nurses visit houses door to door carrying out their services such as antenatal and postnatal care. However, carrying out such services becomes difficult, particularly in those areas where there are no proper concrete roads. It becomes challenging in the monsoon season, where most areas become flooded. Health awareness camps were conducted from time to time to raise awareness of the benefits of modern healthcare services and other preventive measures.

These barriers have been divided into two broad categories based on the service-users’ perspective and the health- service providers’ perspective. Both service users and providers identified that health-service utilization in the study area was hindered by poor health-facility infrastructure, lack of awareness and education among the tribal population, and inadequate public transport and communication. While people’s low economic status, dependence on traditional medicines, and the poor quality of care at government health facilities prevented respondents from using the health facilities effectively, the lack of adequate human resources and government support in health facilities was also implicated.

The study supports findings from previous research that tribal people’s low economic standing is a significant barrier to adequate healthcare utilization [23]. Overall, it was found that 40.6% of the tribal populations lived below the poverty line compared with 20.5% of the non-tribal population in the country [7]. Over two-thirds of the tribal population worked in the primary sector against 43% of the non-tribal population. Tribal people are economically highly dependent on agriculture. Our findings confirm those from previous studies in different contexts that have demonstrated that both direct and indirect healthcare costs and distance deter access to healthcare facilities [24, 25].

Along with weak economic standing, low educational status is an obstacle from both clients’ and providers’ perspectives. Recent NFHS- data for 2015–2016 show that only 6% of tribal women have higher education, as against 19% of the general population. Numerous studies show that education promotes the use of health services via many pathways; for example, by raising awareness of the benefits of using healthcare facilities, more-educated people have a better knowledge of, and access to, facilities or they can negotiate better with health providers [26, 27]. In our study area, the low level of education resulted in poor communication between the patients and service providers [28]. While patients did not fully understand the service providers’ instructions, service providers were also deterred from providing information, as it took more time and effort. Thus the communication gap was another critical barrier between the service providers and the tribal people, leading to low utilization of public healthcare services, as observed in previous studies [29].

Our study further confirms that the poor quality of health care in government facilities in the rural area is still a big challenge, even after implementing several health policies and programs initiated by the government. We found that public facilities still lack human resources and other essential infrastructure; there is the unavailability of medicines, longer waiting times, and poor interpersonal behavior. These findings are also outlined in previous studies [30–32].

Economically vulnerable tribal populations prefer public health facilities due to low hospital costs [19, 33]. However, they fail to obtain good-quality services and are forced to consult private doctors and purchase the medicine from outside dispensaries, beyond their economic capacity [34]. The respondents complained that health providers did not treat them properly and gave them insufficient time [20, 35]. From the service providers’ perspective, they are overburdened with the number of patients each day and cannot give each patient enough time [31, 36].

Another interesting finding of our study is that the traditional, indigenous system of medicine continues to be an essential source of health care among the tribal people in the study area. Several previous studies in India concluded that the tribal people go to the traditional healers before seeking modern medicine [37]. Accessibility and affordability played an important role in seeking traditional medicines among these tribal communities [38, 39].

In rural Assam, we found from both the users and service providers perspectives that poor public transportation and lack of paved roads played a negative role in accessing health facilities. Previous studies observed similar findings [40, 41].

Our study population is doubly disadvantaged in being a marginalized population within India’s underdeveloped state. Therefore, it was essential to collect qualitative data to analyze independent factors that may provide crucial input for further improving these indicators. This study helped us to explore the roles of social, economic, and culture and context in shaping the health care experiences and behavior. Though all issues could not be addressed with this limited study population, the study raises several crucial issues for the broader context. Another limitation is that we were unable to interview people who do not belong to tribal communities so that we could draw comparisons between the experiences of the different groups. Secondly, the study findings represent to our study area and may not be generalizable at the national level.

## Conclusion

The barriers to utilizing health facilities are not always related to a patient’s socio-economic status but also the quality of the health services available and other contextual factors. The perceived dissatisfaction with the quality of care (long queues, lack of equipment, rude behavior) should challenge health care managers to engage in the improvement of service delivery of the health care facilities through structural changes in the institutions. Awareness should be further raised on the value of modern healthcare facilities through various community-level programs. Improving healthcare and transportation infrastructure in rural areas would increase the use of health facilities among these populations. As poor economic status often prevents the tribal population from using modern healthcare services, there should be a promotion of economic activities among the tribal population to raise their socio-economic status. There is a need to improve the communication between tribal population and health workers with the help of tribal leaders and government functionaries. The results of this study highlight where improvements can be directed to increase the use of healthcare facilities in rural areas.

## Supporting information

S1 Appendix List of the health infrastructure and health professionals of Kokrajhar, Baksa, Chirang, and Udalguri districts at the block level in Assam (source: Primary Census Abstract, Census of India, 2011). (DOCX)

S1 Checklist COREQ (Consolidated Criteria for Reporting Qualitative research) checklist. (PDF)

S1 File (PDF)
