# Supplementary material for: A qualitative study of the barriers to utilizing healthcare services among the tribal population in Assam
Source: PLoS One. 2020 Oct 8;15(10):e0240096. doi: 10.1371/journal.pone.0240096 (PMC7544062; doi:10.1371/journal.pone.0240096)
Supplement: S1 Appendix — (DOCX) [file pone.0240096.s001.docx]

**S1 Appendix: List of the health infrastructure and health professionals of Kokrajhar, Baksa, Chirang, and Udalguri districts at the block level in Assam** (Source: Primary Census Abstract, Census of India, 2011)

1) Community health centre

2) Primary health centre (PHC)

3) Primary health sub-centre

4) Maternity and child welfare centre

5) T.B. clinic

6) Hospital-allopathic

7) Hospital-alternative medicine

8) Dispensary

9) Veterinary hospital

10) Mobile health clinic

11) Family welfare centre

12) Medical practitioner (with MBBS Degree)

13) A medical practitioner (with another degree)

14) Medicine shop

15) Others
