# Supplementary material for: A qualitative study of the barriers to utilizing healthcare services among the tribal population in Assam
Source: PLoS One. 2020 Oct 8;15(10):e0240096. doi: 10.1371/journal.pone.0240096 (PMC7544062; doi:10.1371/journal.pone.0240096)
Supplement: S1 File — (PDF) [file pone.0240096.s003.pdf]

6

JAWAHARLAL NEHRU UNIVERSITY
SCHOOL OF SOCIAL SCIENCES

01-03-2018

No. SSS/2018

Office Order No 120/18

Approval of the Dean is hereby conveyed for field work to the following M.Phil./Ph.d student(s) without any financial liability on the part of school for the said purpose, as per University rules, and as detailed below:

S.No.	Name of the student/Deptt.	Period of field work	Place of visit on field trip	Remarks,if any
01	Supriya Singh CSMCH/SSS	01-03-18 to 29-04-18	Adarsh Nagar, Delhi	
02	Shalini Punjabi ZHCES/SSS	11-01-18 to 31-05-18	Delhi	
03	Srishti Chauhan ZHCES/SSS	01-03-18 to 01-05-18	Ambala, Haryana	
04	Bianca Daw ZHCES/SSS	26-02-18 to 30-04-18	New Delhi	
05	Chetana Naskar CSRD/SSS	24-02-18 to 30-05-18	Kolkata, West Bengal	
06	Sanchari Mukhopadhyay CSRD/SSS	23-02-18 to 20-05-18	New Delhi, Haryana, U P & NCR Region	
07	Bandita Boro CSRD/SSS	05-03-18 to 05-04-18	Assam	
08	Aarti Mangal ZHCES/SSS	26-02-18 to 30-04-18	Delhi (Colleges, Central Institute of Education)	
09	Roma Ranu Dash ZHCES/SSS	26-02-18 to 26-04-18	New Delhi	
10	Sreya Sen CSSS/SSS	01-03-18 to 16-03-18	Kolkata	
11	Aadil Farooq CSMCH/SSS	05-03-18 to 21-07-18	Jammu and Kashmir (2 Districts)	

This issues with the approval of the competent authority.

[signature] 1/3/18
(Ashok Kumar)
Assistant Registrar

[initials] 1-3-18

Students concerned.

Copy to:

1. Chairperson CSMCH, ZHCES, CSRD, CSSS — 1768/SSS 09/3/18; 1763/SSS 09/3/18; 1784/SSS 09/3/18; 1755/SSS 09/3/18
2. Office order file
3. Budget file
4. A.F.O. Bills/S&P — 1139/SSS 12-3-2018
